# The roles of bone remodeling in normal hematopoiesis and age-related hematological malignancies

**DOI:** 10.1038/s41413-023-00249-w

**Published:** 2023-03-14

**Authors:** Hengwei Zhang, Jane L. Liesveld, Laura M. Calvi, Brea C. Lipe, Lianping Xing, Michael W. Becker, Edward M. Schwarz, Shu-Chi A. Yeh

**Affiliations:** 1 Center for Musculoskeletal Research, University of Rochester Medical Center, 601 Elmwood Ave, Box 665, Rochester, NY 14642 USA; 2 Department of Pathology and Laboratory Medicine, University of Rochester Medical Center, Rochester, NY USA; 3 Wilmot Cancer Center, University of Rochester Medical Center, Rochester, NY USA; 4 Department of Medicine, Division of Hematology/Oncology and Bone Marrow Transplantation Program, University of Rochester Medical Center, Rochester, NY USA; 5 Department of Medicine, Division of Endocrinology/Metabolism, University of Rochester Medical Center, Rochester, NY USA; 6 Department of Orthopaedics, University of Rochester Medical Center, Rochester, NY USA; 7 Department of Medicine, Division of Allergy/Immunology/Rheumatology, University of Rochester Medical Center, Rochester, NY USA; 8 Department of Biomedical Engineering, University of Rochester, Rochester, NY USA; 9 Department of Physiology/Pharmacology, University of Rochester Medical Center, Rochester, NY USA

**Keywords:** Cancer, Bone, Cancer

## Abstract

Prior research establishing that bone interacts in coordination with the bone marrow microenvironment (BMME) to regulate hematopoietic homeostasis was largely based on analyses of individual bone-associated cell populations. Recent advances in intravital imaging has suggested that the expansion of hematopoietic stem cells (HSCs) and acute myeloid leukemia cells is restricted to bone marrow microdomains during a distinct stage of bone remodeling. These findings indicate that dynamic bone remodeling likely imposes additional heterogeneity within the BMME to yield differential clonal responses. A holistic understanding of the role of bone remodeling in regulating the stem cell niche and how these interactions are altered in age-related hematological malignancies will be critical to the development of novel interventions. To advance this understanding, herein, we provide a synopsis of the cellular and molecular constituents that participate in bone turnover and their known connections to the hematopoietic compartment. Specifically, we elaborate on the coupling between bone remodeling and the BMME in homeostasis and age-related hematological malignancies and after treatment with bone-targeting approaches. We then discuss unresolved questions and ambiguities that remain in the field.

## Introduction

Hematopoietic stem cells (HSCs) reconstitute the blood and immune systems through tightly regulated cell fate decisions that balance self-renewal and multilineage differentiation processes.^1^ Understanding the regulatory mechanisms underlying this balance provides the means to guide emergency hematopoiesis, increase reconstitution capacity after bone marrow transplantation, and intervene in hematologic malignancies.

The stem cell niche concept emerged approximately two decades ago and refers to specialized microenvironments that provide unique functional dimensions to resident cells.^2^ The roles of endosteal and perivascular niches and cell-derived factors in the context of hematopoiesis have been extensively reviewed.^1,3–6^ However, new evidence supports a hypothesis of interdependent skeletal and hematopoietic dysfunction in aging^7^ and hematological malignancies,^8,9^ suggesting that bone, bone marrow, and hematopoietic function needs to be studied as a single unit. In support of a critical role for the skeletal compartment in hematopoiesis, our prior work using intravital microscopy to track native HSCs in the physiological microenvironment revealed that clonal expansion of activated HSCs after cyclophosphamide/granulocyte colony stimulating factor (G-CSF) stimulation was spatially restricted to a subset of bone marrow cavities undergoing bone remodeling and was not evident in cavities predominated by bone formation or resorption alone.^10^ This finding emphasized that dynamic bone turnover acts directly on HSCs and/or, more likely, imposes additional heterogeneity within the bone marrow microenvironment (BMME) to yield differential clonal responses.

**Fig. 1:**
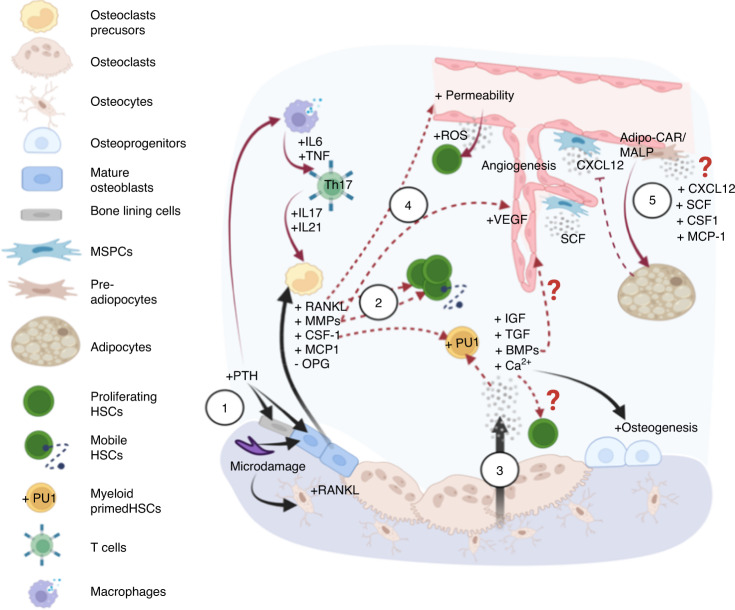
Factors involved in the initiation of bone remodeling and their crosstalk with the hematopoietic compartment. (1) Homeostatic bone remodeling occurs in response to microdamage and systemic Ca^2+^ needs. The nucleating event involves RANKL expression triggered in traumatized osteocytes or by PTH stimulation of bone-lining cells/osteoblasts and immune cells expressing PTH receptors. These lead to production of RANKL and other factors to promote osteoclastogenesis and osteoclast adhesion, respectively. (2) These bone-remodeling cascades exert functional effects on HSCs in the microenvironment via direct stimulation and alterations to the stem cell niche. Examples include myeloid differentiation bias, HSC expansion and mobilization from MCP-1, CSF-1, RANKL and MMPs released from activated osteoblasts. (3) During bone resorption, growth factors and calcium stored in the bone are released to promote osteodifferentiation, which can promote angiogenesis and impact hematopoiesis directly. (4) Global changes to the bone marrow microenvironment can also occur due to RANKL-increased vascular permeability and subsequently elevated ROS levels in HSPCs. (5) Additional changes are mediated by adipocytes and adipocyte-primed progenitors, including a recently identified MALP population that largely overlaps with *LepR*^*+*^ perivascular MSPCs, that release factors important to stem cell maintenance and regeneration (CXCL12 and SCF), myeloid differentiation (CSF-1), and osteoclastogenesis (MCP-1). The overlap in the regulation of homeostatic bone remodeling and hematopoiesis within the same microenvironment leads to several questions to be addressed. The first question pertains to the regulatory mechanisms by which MALP affects HSCs and the bone compartment. The second relates to the need to understand how extracellular calcium modulates HSC dormancy and proliferation. It is also unclear whether the angiogenic effects of growth factors are specific to sinusoidal or arteriolar vessels that are known to exert distinct hematopoietic supports. (In all figures, black solid arrows indicate interactions within the bone compartment; red solid arrows indicate interactions within the hematopoietic compartment; red dashed arrows indicate interactions between the bone and hematopoietic compartments)

**Fig. 2:**
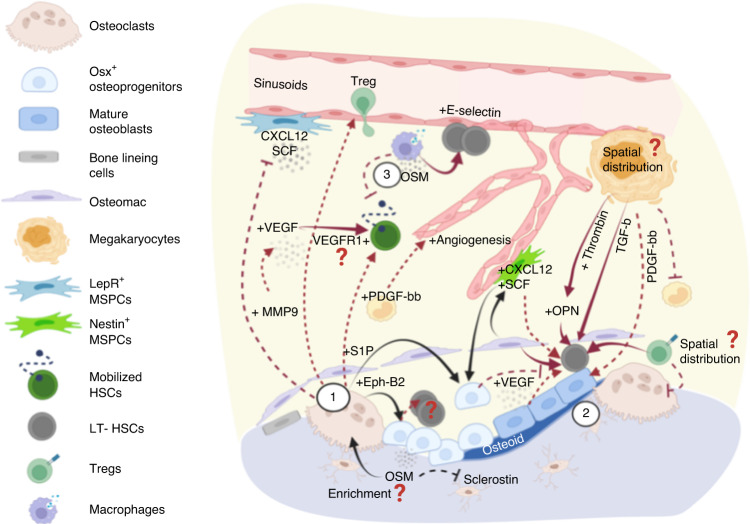
Factors involved in the reversal stage of bone remodeling and their crosstalk with the hematopoietic compartment. (1) Osteoclasts secrete several coupling factors to promote osteodifferentiation during the reversal stage of bone remodeling and that also target hematopoietic cells. S1P acts through S1P_1_ receptors that are highly expressed on hematopoietic cells to regulate cell trafficking, which is crucial for cell egress after treatment with mobilizing agents (e.g., G-CSF or GM-CSF). Osteoclast-derived Ephrin-B2 acts on EphB4-expressing osteolineage cells, leading to the subsequent expansion of long-term HSCs mediated via mechanisms that remain to be elucidated. Osteoclasts also recruit regulatory T cells, which may constitute immune-privileged sites that promote HSC survival. In addition, MMP9 secreted from osteoclasts modulates the bone marrow microenvironment in several ways, including the release of VEGF from extracellular matrix to promote angiogenesis and the degradation/shedding of CXCL12 and SCF to promote HSC mobilization. (2) In a bone remodeling unit, osteolineage cells, osteomacs, and megakaryocytes (MKs) work collaboratively to promote osteogenesis. Each unit produces abundant factors such as thrombin-cleaved, activated OPN, and MK-derived TGF-β, which regulate HSC dormancy. (3) Moreover, oncostatin M (OSM) secreted by osteolineage cells (e.g., MSPCs and osteoblasts) or immune cells (e.g., macrophages) plays pleiotropic roles to promote both remodeling (inducing RANKL expression) and osteodifferentiation (suppressing sclerostin expression). Importantly, OSM induces CXCL12 to inhibit cell mobilization and boost E-selectin-mediated HSC self-renewal/expansion. Overall, the reversal stage involves functionally diverse signaling pathways that promote dormancy, mobilization, and expansion. It will be insightful to understand whether HSC proliferation in such microenvironment is mediated by self-renewal-based expansion or loss of stemness, and whether active bone remodeling leads to enrichment in MK distribution, OPN/OSM concentration, and accumulation of regulatory T cells that are associated with the self-renewal potency and survival of HSCs

**Fig. 3:**
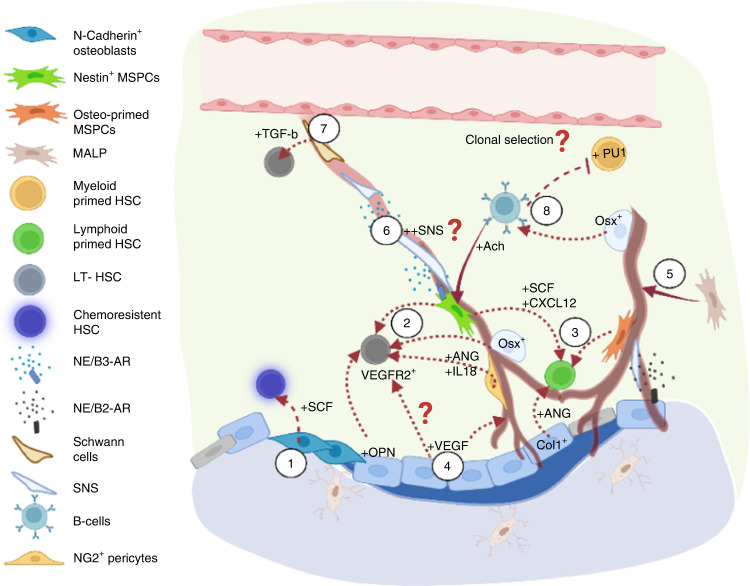
Factors involved in the bone formation stage and their crosstalk with the hematopoietic compartment. (1) N-cadherin^+^ osteoblasts have been shown to be spatially associated with chemoresistant HSCs via SCF regulation. (2) Osteolineage cells maintain phenotypic LT-HSCs via IL-18 and Nestin^+^/Osterix^+^/NG2^+^ cell-derived angiogenin (ANG). (3) In contrast, angiogenin from mature osteoblasts and LepR^+^/osteolectin^+^ osteo-committed MSPCs maintain lymphoid-primed HSCs and common lymphoid progenitors. Periarteriolar Nestin^+^ stromal cells secrete SCF and CXCL12 to maintain lymphoid primed or unbiased HSCs. (4) Osteogenesis impacts the bone marrow microenvironment in several ways. Osteoblast-derived VEGF boosts angiogenesis via VEGFR2, which also promotes the repopulating capacity of HSCs. (5) In addition, osteogenesis is associated with the enrichment of the arteriole-connecting capillaries (Type H vessels), which has been shown to be associated with MALP and Osterix^+^ populations. (6) Moreover, the SNS has been found to be spatially associated with arterioles, which can enhance *Nestin*^*+*^ MSPC-derived CXCL12 secretion through the action of β3-adrenergic receptors and modulate cell motility following circadian cycles. (7) Schwann cells on nerve axons also contribute to HSC quiescence by activating latent TGF-β. (8) During bone formation, *Osterix*^*+*^ osteoprogenitors and osteoblasts are critical for B lymphopoiesis, and pro-B cells secrete acetylcholine to retain HSCs/HSPCs in the bone marrow and suppress the expansion of myeloid cell progenitors. Several questions remain to be answered. For example, do different stages of bone remodeling affect perivascular stromal composition, cytokine gradient, and SNS regulation? Does bone remodeling also shape the spatial landscape of VEGF/VEGFR1^+^ (Fig. 2) and VEGF/VEGFR2^+^ signaling to regulate HSCs differently? Importantly, whether distinct hematopoietic clones exhibit specific tropism toward a given microenvironment during bone remodeling/modeling cycles remains to be addressed

**Fig. 4:**
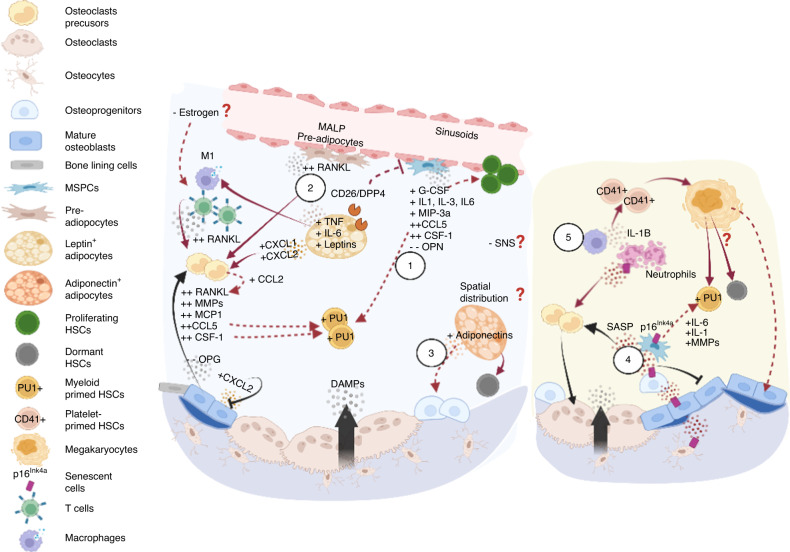
Skeletal aging and its impact on the hematopoietic compartment. Aging of osteolineage cells leads to an overall inflammatory microenvironment (left panel) and acquisition of the senescence-associated secretory phenotype (SASP, right panel). (Left 1) In general, aged osteoprogenitors employ a myeloid-promoting program, attributed to telomere dysfunction that manifests as upregulation of G-CSF, IL-3, MIP-3a levels in bone-associated stromal cells, and aging of bone cells that overexpress CSF-1, IL-1, CCL5, etc. (2) Aged MSPCs are also primed for adipocyte differentiation, synergistically promoting myelopoiesis. Specifically, the preadipocyte (Pref-1^+^ or MALP) population is expanded with upregulated RANKL expression. Adipocytes further secrete leptins, DPP4, IL-6, TNF, and CXCL1/CXCL2 to promote osteoclastogenesis while inhibiting osteodifferentiation, which promotes the proinflammatory immune phenotype. (3) In contrast, adiponectins promote osteoprogenitors and have been shown to protect HSCs from inflammatory insult and enhance self-renewal. Hypothetically, leptin- and adiponectin-secreting adipocytes exhibit spatial associations with bone resorption and formation sites, respectively, and may contribute to differential HSC responses. (Right 4) Multiple cell types, including osteoblasts, osteoprogenitors, osteocytes, lymphocytes, and myeloid cells, undergo senescence and are associated with the overproduction of IL-6, IL-1, MMPs and other proinflammatory cytokines that promote bone resorption and myeloid bias. (5) Reduced phagocytic capability of macrophages leads to the accumulation of senescent neutrophils, an increase in IL-1 level and induced platelet bias. Whether increased megakaryopoiesis causes myeloid bias and HSC accumulation remains unclear. Overall, aging is associated with contraction of Type H vessels, and arterioles, depleting niche factors that support lymphopoiesis and possibly negatively impacting the SNS. Although important, it remains unclear whether effects of estrogen deficiency on bone remodeling kinetics cause substantial variability in aged HSC niches between sexes

**Fig. 5:**
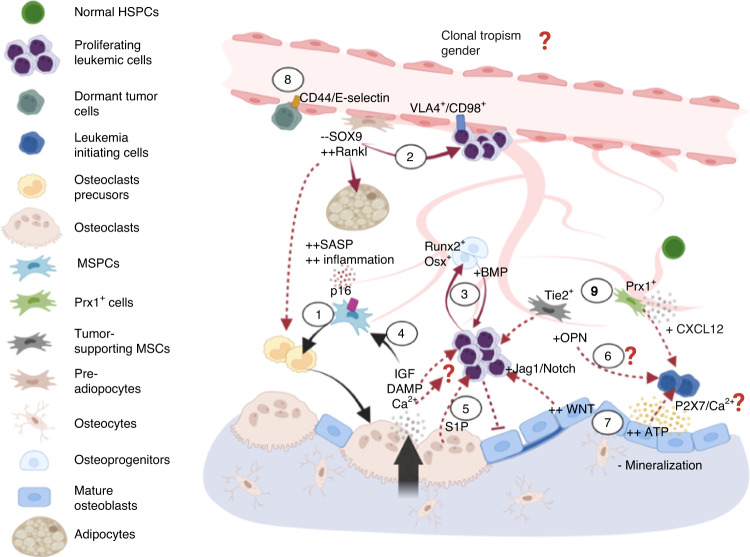
Crosstalk between skeletal aging and age-associated myeloid diseases. (1) MSPC senescence augments the proinflammatory microenvironment (Fig. 4) in MDS/leukemia. (2) An adipogenic propensity promotes the survival of leukemia blasts, although it has been found that (3) in AML, Runx2^+^ and Osterix^+^ osteoprogenitors are expanded via BMP signaling to enhance leukemia engraftment. (4) In addition, growth factors released during bone resorption have been shown to drive SASP acquisition (via IGF) and activate inflammasome NLRP3 (via DAMPs and calcium), which is overexpressed in MDS patients. (5) Coupling factors in the reversal stage may play roles in disease progression. S1P/S1PR_3_ participates in leukemogenesis, myeloid bias and promotes LSC differentiation. In contrast, (6) OPN has been found to drive cell dormancy in B-cell acute lymphoblastic lymphoma; however, its role in MDS and AML still needs to be elucidated. (7) The bone-forming zone may promote leukemia cell expansion and maintenance of leukemia-initiating cells. For example, hyperactive Wnt pathways in osteoblasts have been associated with a differentiation blockade and excessive blast numbers mediated through Jagged1/Notch signaling. Higher ATP levels in the osteoblastic zone sustain leukemia-initiating cells via ATP-P2X7 signaling. Notably, this process involves the influx of calcium ions; however, whether different levels of extracellular calcium at distinct stages of bone remodeling exert differential impacts on leukemic cells remains to be investigated. (8) Distinct subsets in the sinusoidal niche can support the proliferation (VLA-4/CD98) or dormancy (CD44/E-selectin) of leukemia stem cells. (9) CXCL12 from Tie2^+^ periarteriolar stroma has been shown to promote cell proliferation, while CXCL12 from Prx1^+^ MSPCs drives chemoresistance. While both endosteal and vascular niches can support tumor proliferation and dormancy, sex effects (Fig. 4) and whether distinct tumor subclones exhibit tropism toward a given niche remain to be elucidated

**Fig. 6:**
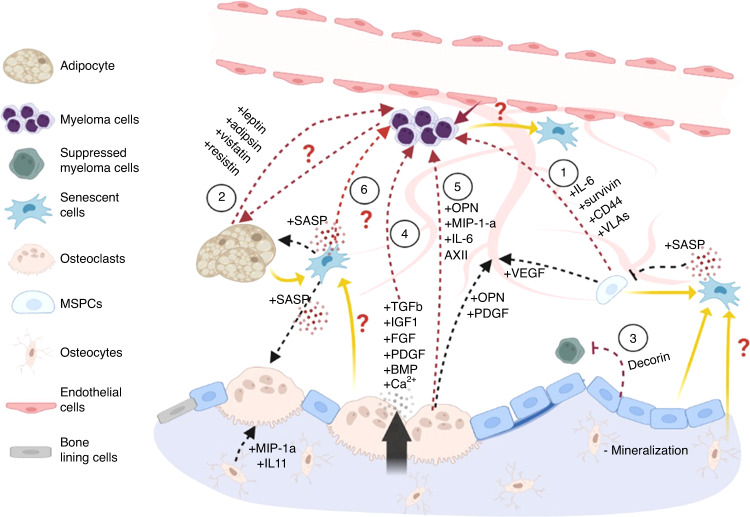
Crosstalk between the components of aged bone and age-associated multiple myeloma. Multiple myeloma is an age-associated disease that is closely related with bone cells. Myeloma cells establish a tumor microenvironment with osteoblastic and osteoclastic cells and generation of bone matrix, which creates a vicious cycle that promotes myeloma cell expansion on bone lesions. The contribution of bone cells and senescent cells under aging conditions to the tumor microenvironment and maintenance of myeloma cells are described as follows: (1) Most cell compartments in the tumor microenvironment, except the osteoblast compartment, support tumors. Mesenchymal progenitors directly promote the proliferation, migration, and adhesion of myeloma cells through the action IL-6, survivin, CD44, VLAs, syndecan 1 and MCP-1 or indirectly affect myeloma cells through osteoclastogenesis and angiogenesis. (2) An increase in the number of adipocytes support MM through their release of adipokines, such as leptin, adipsin, visfatin and resistin. (3) Osteoblasts have been identified as the only cells that inhibit myeloma cells through Decorin. (4) However, osteoblasts indirectly promote myeloma cells through the activation of osteoclasts. Osteoclasts resorb bone matrix, release numerous tumorigenic factors, such as TGF-β, IGF-1, FGF, PDGFs and BMPs, and increase the extracellular calcium level. (5) The direct mechanism by which osteoclasts affect myeloma cells involves the secretion of soluble factors, such as osteopontin, MIP-1a, IL-6, Annexin II, BAFF and APRIL. Although senescent mesenchymal progenitors and adipocytes have been reported in MM, the mechanisms underlying their senescence are still not clear, particularly in the early stages of MM. (6) In MM, an increased number of senescent cells in the tumor environment has been observed, but the contribution of increased senescent cells remains unclear. Senescent cells inhibit osteoblast differentiation but promote osteoclast differentiation through SASP factors

To date, discussions of the “endosteal” niche have been largely focused on the osteogenic components. However, little emphasis has been directed to “dynamic remodeling”, which involves distinct stages of bone resorption, reversal, and deposition cycles. The cellular and molecular mechanisms that govern bone remodeling have been described in great detail by Raggatt, Patridge, and Xie.^11–13^ Given the strong coupling between osteogenesis and angiogenesis,^14–16^ the sympathetic nervous system,^17,18^ and immunity,^19^ bone marrow cavities undergoing various stages of bone remodeling are expected to form distinct BMMEs. Although extensive studies have illustrated coupling between bone turnover and nonskeletal systems, and independent studies have documented interactions between the BMME and HSCs, they have not been consolidated in the context of HSC biology. Therefore, in this review, we summarize the molecular cues and cellular constituents that participate in bone remodeling and are known to interact with the hematopoietic compartment (Fig. 1–3). We also review the impact of skeletal aging on hematopoietic aging (Fig. 4) and age-related blood malignancies (Figs. 5, 6), describe existing bone-targeting approaches, and discuss unresolved questions and ambiguities that remain in the field.

## Coupling of bone remodeling and the HSC niche

### The endosteal niche

The endosteal niche comprises cellular components such as osteoblast lineage cells and osteoclasts and has been deemed critical to the successful engraftment and long-term retention of repopulating HSCs and leukemia-initiating cells, as summarized in separate reviews.^3,5^ Being closely associated with bone, cellular composition, molecular crosstalk, and secreting factors in this niche are tied to distinct phases of bone remodeling, as reviewed below.

#### The activation and bone resorption phases involve pathways associated with HSPC mobilization and myeloid differentiation

At steady state, bone remodeling is activated by microdamage and systemic endocrine factors such as parathyroid hormone (PTH) and estrogen. This signalling triggers a reduction in transforming growth factor-beta (TGF-β) level in osteocytes and activation of PTH-PTH receptor signaling cascades in osteoblasts, initiating osteoclast formation^20,21^ (Fig. 1.1). In this phase, osteoblasts express monocyte chemoattractant protein-1 (MCP-1), also termed CC motif chemokine ligand 2 (CCL2), receptor activator of nuclear factor kappa-B ligand (RANKL) and macrophage colony-stimulating factor (M-CSF/CSF-1), to expand the osteoclast precursor pool and promote the differentiation of functional osteoclasts while reducing the level of antiresorptive osteoprotegerin (OPG), a decoy receptor of RANKL. The biology of RANKL/RANK pathways in bone homeostasis has been summarized well in previous reviews.^22^ Concomitantly, several transcription factors (*e.g*., PU.1, MITF, c-Fos, and NFATc1) are involved in directing myeloid progenitor commitment into functional osteoclasts. Additionally, matrix metalloproteinases (MMPs) secreted by osteoblasts expose the arginylglycylaspartic acid (RGD) adhesion sites on bone to allow osteoclast attachment.^11^

Notably, the aforementioned pathways involved in the activation phase of bone remodeling, including the PTH signaling pathway,^23^ have been reported to participate in hematopoietic functions and are associated mainly with the mobilization and lineage differentiation of hematopoietic stem and progenitor cells (HSPCs) (Fig. 1.2). For instance, CSF-1-Fc has recently been proposed to be a potential mobilization agent, as it enabled the expansion of HSPCs and increases their reconstitution capacity after treatment with G-CSF.^24^ CSF-1 also activated *PU.1* in HSCs, which resulted in myeloid cell differentiation bias.^25^ In addition, CSF-1 expression in a small subset of endothelial cells has recently been shown to form a niche that supports monocytic cell production,^26^ although whether such functional output is regulated directly by CSF-1 remains to be elucidated. Similarly, RANKL is known to preferentially expand/mobilize colony-forming progenitors and Lin^-^/Sca1^+^/c-Kit^+^ (LSK) HSPCs, although it exerts only a slight effect on mature myeloid lineages.^27,28^ Moreover, proteolytic MMPs mediate the bioavailability of a broad spectrum of niche factors, for example, MMPs are involved in the enzymatic degradation of CXC motif chemokine ligand 12 (CXCL12), shedding of surface receptors of the stem cell factor (SCF), and disruption of adhesion molecules, namely, α4β1 integrins and vascular cell adhesion molecule-1 (VCAM1). Therefore, MMPs impact both the biophysical and biochemical cues for HSCs and the regulatory capability of niche cells such as the endothelium and the *Nestin*^*+*^ and *Leptin Receptor*^*+*^ (*LepR*^*+*^) mesenchymal stem and progenitor cells (MSPCs). The role of MMPs in hematopoiesis has recently been comprehensively reviewed.^29^ In general, during bone resorption, the expression of critical niche factors such as SCF and osteopontin was found to be reduced in osteoblasts, and osteoclast-associated proteolytic enzymes (MMP-9 and cathepsin K) inactivate SCF and CXCL12, with cathepsin K further suppressing CXCL12 expression; all these changes lead to HSPC mobilization into peripheral blood^.^^27^

Essentially, bone is a reservoir of growth factors and proteins (Fig. 1.3). Insulin-like growth factor (IGF)-1/2, TGF-β, and bone morphogenetic proteins (BMPs) are released during this stage, and all of these factors are potent regulators of HSC maintenance and are involved in the progression of hematological cancers^30^ (details are presented in Section 2). Notably, bone stores greater than 99% of body calcium which when released by bone resorption mediates subsequent osteogenesis.^31^ Thus, one would expect that bone marrow cells experience substantial changes in the extracellular calcium ([*Ca*^*2+*^]_*e*_) during bone remodeling; nonetheless, our understanding of [*Ca*^*2+*^]_*e*_ regulation in HSC biology is rather limited. It was shown that calcium-sensing receptors are required for the homing of fetal liver HSCs to the endosteum,^32^ indirectly suggesting a role for [*Ca*^*2+*^]_*e*_ in stem cell retention. However, it was also recently demonstrated that [*Ca*^*2+*^]_*e*_ that is at least 10-fold lower than the serum calcium concentration plays a greater role in sustaining phenotypic HSCs in vitro.^33^ By quantifying the [*Ca*^*2+*^]_*e*_ in vivo at single-cell resolution, we recently revealed that [*Ca*^*2+*^]_*e*_ was, indeed, tied to local bone remodeling activity and increased with age.^34^ Intriguingly, in contrast to the in vitro finding, we found preferential localization of HSCs in regions in which the [*Ca*^*2+*^]_*e*_ was significantly higher than the serum calcium concentration.^34^ Taken together, these findings further suggest that the availability of secreted niche factors and [Ca^2+^]_e_ in the endosteal microenvironment is altered at distinct phases of bone remodeling. How these chemical gradients govern the functional heterogeneity of HSCs (e.g. quiescence or activation) in vivo remains to be investigated.

#### The reversal and bone formation phases are associated with the self-renewal of HSCs and lymphopoiesis

In the reversal stage, osteoclasts secrete coupling factors to promote osteodifferentiation^11^ (Fig. 2.1). Their impact on hematopoiesis has been revealed: In mice in which osteoclasts showed compromised proton production (*oc/oc* mice), HSC homing to the bone marrow was inefficient due to a reduction in osteolineage differentiation.^35^ Specifically, the coupling factor sphingosine 1-phosphate (S1P) has been shown to positively regulate HSPC mobilization. It plays crucial roles in inflammation, as its receptor (S1PR) is highly expressed on hematopoietic cells and has been shown to regulate cell trafficking, immune cell responses and vascular integrity.^36^ Disruption of the S1P/S1PR_1_ axis via pharmaceutical (FTY720) or genetic (*S1P*_*1*_^-/-^) treatment resulted in impaired cell egress after treatment with a chemokine receptor CXCR4 antagonist (AMD3100). Interestingly, disruption of S1P activity also compromised the reconstitution capacity of mobilized donor cells, suggesting that S1P signaling participates in the egress of primitive HSCs with long-term repopulation ability.^37^

In addition to the effect of S1P, osteoclast-derived Ephrin-B2 acts on Eph receptor B4 (EphB4)-expressing osteolineage cells to facilitate bone formation, with EphB4 forward feeding signals to Ephrin-B2-expressing hematopoietic cells to inhibit osteoclast differentiation.^38^ Notably, Ephrin-B2 is highly enriched in HSCs. Overexpression of EphB4 in *Col1*^*+*^ cells expanded the phenotypically long-term HSCs (LSK/CD150^+^/CD48^-^) and increased the bone marrow reconstitution rate, suggesting its role in HSC maintenance.^39^ As Ephrin-B2/EphB4 interactions depend on cell contact, it raises questions of whether promoting the coupling of osteoclasts and osteoblasts creates a local microenvironment that enhances the self-renewal of HSCs. Consistent with this speculation, intermittent PTH treatment increased osteoblast and osteoclast interactions^40^ and has been shown to expand HSCs and improve engraftment.^41^ While this coupling process is compromised in older patients, manipulating the coupling factors involved during the reversal stage of bone remodeling may synergistically support HSC expansion (EphrinB2/EphB4) and mobilization (S1P/S1PR_1_) during bone marrow transplantation.

In addition to osteoclast-derived coupling factors, osteolineage cells, bone resident macrophages (F40/80^+^/TRAP^-^ osteomacs^42^), and megakaryocytes (MKs) work collaboratively to initiate osteogenesis (Fig. 2.2). Deletion of CD169^+^ macrophages resulted in loss of mature osteoblasts and compromised both intramembranous and endochondral bone repair.^43^ In vitro, inhibition of osteoclastogenesis and an increase in the osteoblast proliferation rate have been observed when bone marrow macrophages or a murine preosteoblast line was cocultured in MK-conditioned medium.^44^ In addition, MKs secrete platelet-derived growth factor-BB (PDGF-BB), which promotes the recovery of osteoblasts after total body irradiation.^45^ Notably, osteolineage cells, and potentially osteomacs are sources of OPN,^42,46^ a glycoprotein crucial in guiding the osteogenic differentiation of MSPCs^47^ and essential in enhancing HSC engraftment^48^ and limiting the HSC pool size.^49^ Specifically, thrombin-cleaved OPN allows HSC binding to α4β1 (VLA-4) and α9β1 integrins, driving them into a dormant state.^50^ In agreement, studies with OPN-KO mice showed a twofold increase in HSC numbers and acquisition of aging phenotypes, as indicated by increased myeloid bias and decreased lineage reconstitution capacity.^49,51^ Notably, the MK population is a main source of thrombin,^52^ suggesting a potential spatial gradient of thrombin-cleaved OPN fragments concentrated near MKs at remodeling sites, which may lead to the heterogeneous fate determination of HSCs. This postulation may be supported by additional evidence showing roles for MKs in regulating HSC maintenance and stress responses: MKs were found to be spatially associated with HSCs and to regulate cell quiescence via the action of CXCL4,^53^ TGF-β1,^54^ and thrombopoietin.^55^ The MK population also regulates HSC proliferation under stress conditions via fibroblast growth factor (FGF)-1.^54^ Moreover, in a coculture setting, close interactions of osteomacs, MKs and an osteoblast network were shown to increase the number of HSC colony forming units via the regulation of CD166 and embigin.^56^ Taken together, these reports suggest that, although it has not been explicitly demonstrated by imaging, osteomacs, MKs and osteoblasts are likely spatially associated and positively regulate HSC self-renewal and lodging in the endosteum (Fig. 2.2).

Several studies have revealed the regulatory roles of osteoblasts in HSC function in the bone formation phase. For example, long-term HSCs (CD45^+^/Lin^−^/BrdU^+^ cells) appeared to be spatially associated with spindle-shaped N-cadherin^+^ osteoblasts (Fig. 3.1). Inactivating the BMP receptor type 1a (BMPR1a) expanded the osteoblastic niche and the HSC number^57^ Interestingly, chemoresistant HSCs (Lin^−^/CD48^−^/CD41^−^/CD150^+^/CD49b^−^ cells), likely a subset of long-term HSCs, have also been found to be closely associated with N-cadherin^+^ bone and stromal cells. Conditional deletion of SCF in a N-cadherin^+^ population significantly reduced the repopulating capacity of HSCs, especially under chemotherapy-induced stress conditions.^58^ Moreover, Silberstein et al. showed that long-term HSCs were maintained by osteolineage cells that expressed high levels of *IL-18*, *angiogenin*, and *Embigin* (Fig. 3.2). Deletion of *angiogenin* in *Nestin*^+^ MSPCs, *Osterix*^+^ osteoprogenitors, and *NG2*^*+*^ periarteriolar cells resulted in an increased number of cycling HSC long-term HSCs and compromised multilineage reconstitution. In contrast, *Angiogenin* deletion from *Col1a1*^+^ osteoblasts increased the cycling activity of common lymphoid progenitors and lymphoid reconstitution defects after competitive transplantation, suggesting a regulatory role for mature osteoblasts in lymphopoiesis.^59^ This hypothesis is supported by the recent finding indicating that mechanically induced bone formation maintains the population of lymphoid-primed progenitors^60^ (Fig. 3.3). This outcome is also consistent with the known role of osteoblast lineage cells in supporting B lymphopoiesis via the osteoblast-specific heterotrimeric G protein alpha subunit.^61,62^ Additionally, PTH/PTH receptor signaling through *Osterix*^*+*^ osteoprogenitors has been found to be critical for B-cell precursor differentiation and mobilization of mature B lymphocytes.^63^ Green et al. reported recently that B lymphopoiesis was specifically supported by a subpopulation of skeletal lineages (PDGFR*α*^+^/PDGFRβ^+^ cells) with *LepR*-MSPC gene signatures and that mainly localize in trabecular bone-enriched regions.^64^ Taken together, bone-forming sites include cell populations and signaling pathways to support long-term retention of quiescent HSCs and lymphopoiesis. The distinct osteolineage populations and their restricted locations that support lymphopoiesis speaks to an important emerging perspective on the spatial and functional heterogeneity of osteolineage cells.

### The perivascular niche

The perivascular niche comprises heterogeneous groups of endothelial and perivascular stromal networks that regulate HSC retention, self-renewal, and trafficking via several pathways (*e.g*., Notch,^65^ E-selectins,^66^ CXCL12 and SCF^4,17,67–71^), as reviewed comprehensively by multiple research groups.^1,72,73^ With advances in high-resolution imaging and lineage identification protocols, increasing evidence suggests substantial heterogeneity within the perivascular niche that regulates the lineage commitment of hematopoietic cells.^26,60,69,71^

As vascularization is tightly coupled to bone development and regeneration via vascular endothelial growth factors (VEGF) and angiocrine factors,^74–76^ distinct vascular compositions have been spatially associated with osteogenesis.^14^ Here, we review the effects of bone turnover on vascular remodeling with a particular focus on the spatiotemporal variation in the vascular landscape and the perivascular niche in bone marrow.

#### The activation and bone resorption pathways involved in regulating vascular permeability, angiogenesis, and HSC mobilization

During the activation phase of bone remodeling, the RANKL–RANK interaction on endothelial cells has been shown to increase vascular permeability and angiogenesis through TRAF6-PI3K-AKT-dependent pathways.^77^ In turn, an increase in vascular permeability can elevate the level of reactive oxygen species (ROS) in HSPCs, which promotes cell mobilization and suppresses the long-term repopulating capacity of HSCs^78,79^ (Fig. 1.4).

MMPs released during the resorption phase also impact the perivascular microenvironment. For example, enzymatic activities of MMP9 release VEGF from the extracellular matrix, which activates endothelial cell proliferation (Figs. 1.4, 2.1). Roodman et al. showed that arteriolar angiogenesis was abrogated in Mmp9^-/-^ mice and rescued by exogenous VEGF treatment, suggesting the positive regulation of angiogenesis by osteoclast-derived MMPs.^80^ Synergistically, BMP2 released from bone is a potent activator of endothelial cell migration and angiogenesis^81^; however, whether the angiogenic effects differ in arteriolar and sinusoidal vascular subsets, which may create spatiotemporal heterogeneity following bone remodeling, remains unclear. The angiogenic factors then interact with VEGF receptor 1 (VEGFR1) expressed on HSCs to promote mobilization^82^ (Fig. 2.1). Similar to their effects on the endosteal niche, MMPs reduce the availability of HSC maintenance factors produced by the perivascular niche, by inducing SCF shedding^83^ and CXCL12 degradation^84^, thereby compromising cell self-renewal and promoting HSPC motility (Fig. 2.1). Notably, MMPs may drive HSC quiescence by releasing TGF-β produced by MKs and Schwann cells.^29^ In other words, the cell constituents surrounding HSCs likely determine the cytokine and growth factor composition despite having the same enzymatic regulations from MMPs .

#### The reversal and bone formation phases promote the periarteriolar niche and HSC maintenance

Osteoblast-derived VEGF is critical to the ossification process and bone healing^85,86^, by inducing the migration and proliferation of endothelial cells through the action of VEGF receptor 2 (VEGFR2), a vital receptor in both angiogenesis and hematopoiesis. Blocking VEGFR2 with the monoclonal antibody 1C11 has been shown to reduce the number of HSPCs and impair the repopulating capacity of HSCs,^65^ suggesting that VEGF/endothelial VEGFR2 promotes the acquisition of a stem cell phenotype. Thus, VEGF availability during physiological bone remodeling can impact HSCs through either VEGFR1 (Fig. 2.1) and VEGFR2 (Fig. 3.4) to affect HSC motility and maintenance, respectively. However, whether VEGFR1^+^ and VEGFR2^+^ HSCs are differentially distributed and whether expression of VEGFR1^+^ or VEGFR2^+^ are regulated by bone remodeling remain unclear.

Structurally, arteriole-connecting Type H capillaries are spatially correlated with *Osterix*^*+*^ osteoprogenitors in long bones^14^ and in calvarial bones.^87^ Thus, microregions undergoing osteodifferentiation can be expected to create a unique endothelial/perivascular stromal landscape and cytokine composition.^88^ These differences can differentially impact the hematopoietic compartment, as the periarteriolar niche has been reported to constitute distinct regulatory mechanisms from those involved in the perisinusoidal niche.^89,90^ For example, SCF secreted by *Sca1*^bright^/PDPN^-89^ and CXCL12 secreted from *Tie2*^+^ arteriolar endothelial cells have been shown to maintain the HSC number and reconstitution capacity, and to promote HSC recovery after myeloablation.^90^ Deleting periarteriolar *Nestin*^+^ MSPCs reduced both short-term (LSK) and long-term (SLAM) HSCs with an increase in cell egress.^17^ Deletion of *Cxcl12* from NG2^+^ periarteriolar cells altered the periarteriolar localization of the HSC subset.^88^ Notably, arterioles were found to be crucial in supporting *Vwf*^*−*^ lymphoid or unbiased HSC differentiation,^70^ consistent with a recent finding showing that osteogenic *Osteolectin*^*+*^*/LepR*^*+*^ periarteriolar MSPCs were critical for maintenance of lymphoid-biased progenitors^60^ (Fig. 3.3).

In contrast, regions enriched with sinusoidal vessels likely form distinct microenvironments with various cellular and noncellular constituents (*e.g*., enrichment with adipogenic stroma, MKs, and *Vwf*^*+*^ myeloid-biased HSCs^70^, adhesion molecules such as E-selectins^66^ and different composition of chemokines/cytokines). This functional heterogeneity is now better understood with single-cell transcriptome studies. Tikhonova et al. showed that sinusoidal vessels were surrounded by *LepR*^*+*^ MSPC subsets (*Mgp*^high^ or *Lpl*^high^) that showed adipogenic tendencies and were enriched with critical niche factors that support hematopoiesis and myeloid lineages (*e.g*., CXCL12, SCF, IL-7, CSF-1, and MCP-1).^91^ The adipogenic subcluster has also been shown to expand after 5-fluorouracil challenge or irradiation to promote HSC regeneration via the secretion of SCF.^92^ Moreover, Qin and colleagues recently identified a novel population (marrow adipogenic lineage precursors, MALPs) that forms a dense 3D network in marrow, with the majority of it composed of *LepR*^+^ cells^93^. These cells are critical for supporting bone and vascular niches and express key HSC niche factors such as CXCL12 and SCF (Fig. 1.5, 3.5), but how they fit within known MSPC subsets^94^ and how they impact HSCs remain to be explicitly demonstrated. Overall, while the periarteriolar and perisinusoidal niches contribute to a large overlap among cytokines, whether the two niches mobilize distinct fractions/subsets of hematopoietic cells and whether the mechanisms of action are coordinated remain to be determined.

Finally, oncostatin M (OSM) is a cytokine secreted by osteocytes, osteoblasts, macrophages, and T cells. As reviewed by Sims et al., it is known to play pleiotropic roles in coupling bone resorption and formation processes by driving RANKL production in osteoblasts (promoting bone resorption); at the same time, stimulating osteogenesis by suppressing osteocyte-derived sclerostin expression and priming MSPCs for osteoblast differentiation.^95^ In other words, OSM may be the most concentrated in bone marrow locations with active bone turnover, especially during the transition phase leading to bone resorption or bone formation. From the hematopoietic perspective, OSM has recently been shown to control HSC responses to mobilization agents. It functions by enhancing CXCL12-mediated chemotaxis and endothelial E-selectin-mediated HSC proliferation^96^ (Fig. 2.3). These findings imply a high degree of OSM-induced regulation near perisinusoidal (E-selectin^+^) zones under the condition of active bone remodeling and represent a unique example showing that bone turnover affects the microenvironment to modulate the HSC population.

In summary, sites of bone formation constitute periarteriolar rich microenvironments associated with lymphopoiesis, in contrast to the myeloid-supportive perisinusoidal niches. Notably, vascular permeability (in turn, altering ROS levels), perivascular stromal composition, and cell-derived factors are likely different as bone marrow cavities undergoing different stages of bone turnover. Whether distinct hematopoietic clones generated during homeostasis or malignancies exhibit specific tropism toward a given vascular subset is unknown and represents an important line of inquiry.

### Bone remodeling impacts the landscapes of the sympathetic nervous system and Schwann cells that regulate circadian HSC mobilization and maintenance

The regulation of the central nervous system (CNS) and its effect on bone homeostasis through leptin, serotonin, and neuropeptide Y receptors has been clearly illustrated by Maryanovich and Elefteriou.^18,97^ In particular, β-adrenergic signals negatively regulate bone mass through the action of osteoblasts to produce RANKL and promote osteoclastogenesis.^98^ In contrast, signaling from the parasympathetic nervous system positively regulates bone mass by activating nicotinic receptors on osteoclasts, which subsequently induces apoptotic cell death and osteoblast proliferation.^99^ These results suggest the involvement of CNS activities at bone marrow sites undergoing active remodeling, either at the reversal stage or before activation of bone resorption.

Indeed, numerous previous findings have implied that the spatial heterogeneity of bone remodeling stages can affect the SNS and Schwann cell landscape in bone marrow. Specifically, the sympathetic nervous system (SNS) has been found to be spatially associated with the arterioles (enriched in bone formation sites, Section 1.2), where it regulates hematopoiesis in numerous ways. For example, it targets CXCL12 production in *Nestin*^*+*^ MSPCs mediated through β3-adrenergic receptors (Fig. 3.6), modulating cyclic HSC mobilization and egress in response to circadian cycles.^17,100–102^ SNS has also been shown to enhance hematopoietic reconstitution by promoting the survival of *Nestin*^*+*^ MSPCs and CD31^+^ endothelial cells after genotoxic injury, consistent with the observation that arterioles are affected to a lesser degree by genotoxic conditioning.^103^ Notably, sympathetic tone can be activated by OPN, which is produced abundantly during the reversal stage of bone remodeling, and by osteoblasts.^104^ Therefore, hypothetically, sympathetic tone is higher in bone marrow cavities in the reversal stage or during osteogenesis, which may alter HSC mobilization and local bone marrow recovery. In addition to the SNS, Schwann cells have been shown to support angiogenesis and osteogenesis in a coculture system.^105^ Approximately one-fifth of CD150^+^/CD48^-^ HSCs in 2D bone marrow sections were found to be in direct contact with the nonmyelinating Schwann cells on nerve axons, and this contact was associated with HSC quiescence mediated via the activation of latent TGF-β^106^ (Fig. 3.7). Interestingly, this spatial association with Schwann cells was higher than that with osteocalcin^+^ osteoblasts (13.3%) and was found only among HSCs, not in short-term progenitors (LSKs).

Given the potential association of bone remodeling and the central nervous system, whether the osteoporotic phenotype in an aged or malignant microenvironment (Section 2) exacerbates SNS and Schwann cell degeneration and hematopoietic incompetence remains to be determined.

### Bone remodeling characterizes immune cell distribution and pro- or anti-inflammatory phenotypes

Immune cells participate in bone homeostasis at steady state and in blood malignancies, which have been extensively explained in recent reviews on osteoimmunology and are therefore not elaborated in this review.^19,107,108^ However, it is worth noting that in ovariectomized mouse models of menopause, the CD40 ligand of T cells was shown to promote the osteoblastic differentiation of CD40-expressing MSPCs while simultaneously upregulating osteoclastogenic activities by enhancing M-CSF and RANKL expression.^109^ These outcomes imply increased bone turnover in aged females, which is further discussed in Section 2. In the following section, we review factors involved in each stage of bone remodeling that also play significant roles in regulating immune cell activities and cytokine secretion.

#### The activation and resorption phases promote proinflammatory phenotypes

PTH receptors are expressed not only by osteolineage cells but also by T cells.^110^ Therefore, after stimulation with PTH, both bone and immune cells release interleukin (IL)-6, TGF-β and tumor necrosis factor (TNF)-*α*, to promote differentiation of the proinflammatory Th17 T-cell subset and increase the levels of proinflammatory cytokines such as IL-17, IL-21, and IL-22^111^ (Fig. 1.1). A inflammatory phenotype plays critical roles in regulating HSCs and aged hematopoiesis, which is further elaborated in Section 2.

#### Distinct subsets of immune cells are recruited during the reversal and formation phases

Osteoclasts recruit CD8^+^/CD25^+^ T cells and regulatory T cells (Tregs) to blunt bone resorption, and CD4^+^/CD25^+^/*Foxp3*^+^ Tregs inhibit osteoclast formation by suppressing CSF-1 and RANKL expression (Fig. 2.1). Notably, these processes are cell-contact dependent and mediated by CTLA-4 (CD152), as the separation of Tregs from osteoclast precursors in vitro completely abolished the antiresorption effects of the Tregs.^112^ In an important connection to hematopoiesis, Tregs in the endosteal zone protect HSCs from immune attack and allow cell survival after allogeneic transplantation without immunosuppression.^113^ Thus, understanding whether bone remodeling shapes a distinct spatial distribution of Tregs or "immune-privileged sites" may provide critical insight into the modulation of immune responses during bone marrow transplantation. In contrast, the coupling factors at the reversal stage of bone remodeling may also regulate the distribution of proinflammatory immune cells in a S1P and OPN signaling-dependent manner. Specifically, surface expression of S1P_1_ receptors on innate immune cells and lymphocytes regulate the migratory behaviors of these cells toward inflammatory sites and secondary lymphoid organs, respectively.^114^ Moreover, OPN induces the survival, differentiation and retention of multiple immune cell lineages, such as monocytes/macrophages, via interactions with integrins, CD44,^115^ and dendritic cells.^116^

As described above, *Osterix*^*+*^ osteoprogenitors and osteoblasts are critical for B lymphopoiesis,^117^ suggesting that B lymphopoiesis may be spatially associated with bone formation sites and, potentially, Type H vessels. Notably, it has been recently shown that pro-B-cell-derived acetylcholine served as a parasympathetic regulator in bone marrow to retain phenotypic HSCs and LSKs and inhibit the expansion of common myeloid progenitors (Fig. 3.8). The responder stromal cells marked by *Chrna7* expression were found to be highly enriched in arteriolar subsets,^118^ consistent with the observation of limited clonal expansion in bone deposition sites.^10^

Taken together, these data suggest that dynamic bone remodeling can be expected to define the immune landscape and cytokine gradients. A better understanding of these relationships and their functional impacts on HSCs may provide additional insight into potential immunotherapies and means to modulate immune responses under stressed hematopoiesis.

## The role played by bone remodeling in aging and aging-related hematological malignancies

With increasing age, bone remodeling is gradually biased toward net loss due to functional aberrations and the fate drift of osteolineage cells, as well as an increase in proinflammatory factors that further accelerate bone resorption. In addition to skeletal aging, hematopoietic aging increases the risk for infection and anemia. Overall, the number of HSCs increases but is accompanied by functional decline, including reduced self-renewal and homing capacities, and increased myeloid/platelet bias at the expense of reduced lymphoid/erythroid cell output.^6^ While cell-intrinsic factors contribute to hematopoietic aging, how the bone marrow microenvironment augments or potentially reverses the aging phenotype remains under intense investigation. Increasing evidence suggests that aged HSCs, when placed in a young microenvironment, restore lymphoid commitment,^119^ supporting a corrective role from the microenvironment. Herein, we focus on functional aberrations of the bone compartment during aging and their impact on aged hematopoiesis (Section 2.1) and aging-related hematological malignancies (Sections 2.2-2.3).

### Skeletal aging and the bone marrow microenvironment

The overall osteoporotic phenotype can be attributed to several distinct yet interdependent factors involving telomere dysfunction, cell senescence, differentiation bias toward adipocytes, low-level inflammation, and estrogen deficiency. In Section 2.1, we review the mechanisms of skeletal aging and how aged bone affects the hematopoietic microenvironment and function.

#### Telomere dysfunction impairs MSPCs and facilitates myeloid bias

Numerous studies have suggested an association between telomere shortening and osteoporosis in patients, as recently reviewed.^120^ In a mouse model of accelerated aging that exhibited shortened telomeres (*Wrn*^*-/-*^*, Terc*^*-/-*^ double mutant), MSPCs exhibited impaired osteogenic potential in vitro with negligible staining of alkaline phosphatase and Alizarin, which was accompanied by a marked decrease in OPN secretion from bone marrow MSPCs in mice that were 10 months of age.^121^ Notably, telomere dysfunction in aged MSPCs impairs hematopoiesis. Studying telomerase-deficient (*Terc*^-/-^) mice, Ju et al. showed age-dependent upregulation of G-CSF, a B-cell chemoattractant, IL-3, and macrophage inflammatory protein (MIP)-3a in bone-associated MSPCs, which impaired HSC engraftment^122^ (Fig. 4.1). Specifically, healthy bone marrow cells transplanted into sublethally irradiated aged *Terc*^-/-^ mice led to impaired B lymphopoiesis and myeloid bias, but this phenotype was rescued when *Terc*^*-/-*^ bone marrow cells were transplanted into healthy recipients, confirming the regulatory role of dysfunctional MSPCs in the hematopoietic compartment.^122^

#### Adipogenic predisposition modulates HSC recovery and self-renewal through inflammatory programs and adipokines

Increased microRNA-188^123^ and RANKL levels have been associated with adipocyte commitment and expansion of RANKL^+^ preadipocytes (*Pref-1*^+^) in the aging context. In addition, Zhong et al. performed single-cell transcriptome analyses for the mesenchymal lineage cells, in which MALPs had been identified (Section 1) and shown to express high levels of RANKL and expand during aging.^93,124,125^ As shown in Fig. 4.2, RANKL^+^ preadipocytes, together with adipocytes that produce IL-6 and TNF, altered the RANKL/RANK/OPG balance in favor of osteoclastogenesis. Adipogenic cells also overproduced CXCL1/CXCL2^126^ and DPP4^127^ which suppressed *Runx/Osterix*^*+*^ osteoprogenitors and osteodifferentiation. These impacts on bone remodeling are thought to influence the bone marrow microenvironment, as discussed in Section 1. Moreover, adipokines (factors secreted by adipocytes), such as lectins and adiponectins, are associated with skeletal and immune modulation, as described in a recent review.^128,129^ For example, leptins not only act on skeletal stem cells to shift their fate to adipogenesis under a diet-based stimulation^130^ but also enhance proinflammatory programs, such as the secretion of IL-6 from monocytes, chemokines from macrophages, and Thy1 from polarized CD4^+^ T cells. Adipocytes have also been shown to switch macrophages to the proinflammatory phenotype in obese mice.^131^ In contrast, adiponectins play roles in guiding the migration and differentiation of osteoprogenitors (Fig. 4.3) to resorptive regions or sites of microdamage.^128^ In other words, the adipocyte population can regulate hematopoiesis through the microenvironment and potentially through bone remodeling.

Adipocytes also impact hematopoiesis directly in numerous ways; however, whether they play beneficial or detrimental roles remains controversial in part due to the different effects they exert on stem or progenitor cells and differences in the locations of the adipose tissue. For example, depleting adipocytes in fat-enriched tail vertebrae increased the expansion of progenitor cells after irradiation, while this effect was less prominent in the stem cell (LSK) compartment.^132,133^ With a positive regulatory effect, adipocytes in long bones has been shown to promote the regeneration of HSCs via increased SCF secretion after exposure to radiation-induced stress.^92^ Interestingly, Meacham et al. demonstrated a role for adiponectin in maintaining HSC self-renewal throughout aging and protecting HSCs from insults caused by inflammatory cytokines.^134^ Notably, these effects were specific to HSCs and less prominent in differentiated cells.

Overall, these studies suggest that adipocytes regulate hematopoiesis through both direct and indirect mechanisms. Given the positive role of adiponectins in bone formation, one can postulate that osteogenic sites may exhibit a high local concentration of adiponectins that may favor HSC self-renewal. Future work is required to better understand whether bone marrow cavities that undergo different stages of bone remodeling harbor functionally different adipocytic subsets and how this variability modulates inflammatory responses, hematopoietic recovery, and HSC self-renewal.

#### Replicative senescence reinforces an inflammatory microenvironment

In aged animals, multiple sets of cells in the bone marrow microenvironment exhibit a 5- to 10-fold increase in the level of p16^Ink4a^, a marker of cellular senescence. These cells include osteoblasts, osteoprogenitors, osteocytes, B and T lymphocytes, and myeloid cells, of which osteoprogenitors, myeloid cells and osteocytes constitute the key populations critical for acquisition of the senescence-associated secretory phenotype (SASP), which is associated with excessive production of IL-6, IL-1, proinflammatory cytokines/chemokines, and MMPs^135^ (Fig. 4.4). The inflammatory milieu thus promotes osteoclastogenesis and inhibits osteoblast differentiation and mineralization. The effects of inflammation on skeletal and hematopoietic functions is discussed below.

#### The inflammatory phenotype promotes myeloid bias and compromises HSC self-renewal

As shown in Fig. 4.1–4.4, largely because of intrinsic skeletal aging,^7,136^ the bone marrow microenvironment is characterized by increased levels of proinflammatory cytokines. For example, the age-associated inflammatory phenotype in stromal cells (*e.g*., expression of IL-1β, IL-6, CCL5, etc.) overlaps significantly with the transcriptome signatures induced by lipopolysaccharide (LPS) and pl:C which mimic bacterial and viral infections, respectively.^137^ Notably, the transcriptional profiles of aged skeletal stem cells were found to be switched from osteo- or chondrogenic to pro-myeloid programs, as manifested by a profound increase in CSF-1 expression and systemic elevation of serum IL-1β, IL-10, and TNF levels.^7^ These proinflammatory factors have also been shown to expand a highly active osteoclast population derived from the Ly6C^high^/CD11b^high^ cells.^138^ Among these proinflammatory factors, IL-1 has been suggested to play a significant role in hematopoietic aging and myeloid malignancies. Long-term exposure of HSCs to IL-1 led to myeloid bias and reduced HSC self-renewal capacity.^139^ Moreover, it has recently been shown that the reduced phagocytic capability of aged macrophages resulted in the accumulation of senescent neutrophils in bone marrow, which contributed to increased IL-1β and platelet bias^140^ (Fig. 4.5).^140^ Other proinflammatory factors, such as IL-6 or TNF, were reported to activate mTOR in HSCs, compromising their reconstitution capacity.^141^

Aging also involves a linear increase in pro-inflammatory chemokines crucial to both bone remodeling and a myeloid cell differentiation program. CCL2 (MCP-1) produced by preosteoclasts positively regulates RANK expression and promotes bone resorption. It is also a chemoattractant, recruiting CCR2^+^ monocytes, macrophages, and HSPCs to inflammation sites.^142^ In contrast, CCL5 has been shown to promote the survival of osteoblasts^143^ and is known to stimulate myeloid cell differentiation^144^ via the transcription factor *Gata2* and to promote hematopoietic regeneration after irradiation^145^ (Fig. 4.1). Notably, CCL2 and CCL5 exert opposite effects on bone remodeling; however, why CCL5 does not lead to bone anabolism is unclear.

#### The endocrine phenotype further characterizes skeletal aging and immunity

Estrogen receptors are present on both bone and immune cells. Estrogen deficiency in postmenopausal women is associated with T-cell expansion, excess production of inflammatory cytokines, and osteoclastogenesis.^146^ Notably, telomere shortening is more consistently observed in females than in males. Aged females may also manifest accelerated turnover. Taken together, sex differences likely cause substantial variability in bone remodeling kinetics, impacting HSC niches (Section 1), and should be considered in studying age-related hematological disorders.

#### Skeletal aging compromises the endosteal perivascular niche critical to HSC maintenance and lymphpoiesis

Distinct vascular subsets are coupled to osteogenesis; therefore, alteration in the bone compartment during aging is thought to alter the vascular architecture and dysregulate the perivascular niche. For example, preosteoclast-derived PDGF-BB promotes angiogenesis of Type H vessels (CD31^+^, EMNC^+^) to couple the bone resorption–formation process (Fig. 2.1). However, ovariectomized mice that mimic osteoporosis exhibited reduced PDGF-BB secretion and Type-H vessels, while the phenotype was rescued by increasing PDGF-BB via local or systemic cathepsin K inhibition.^147^ In agreement with this finding, the number of *Nestin*^*+*^ arterioles and Type-H vessels was found to decrease with aging,^14,148,149^ and this change was accompanied by marked decreases in endothelial Notch signaling,^149^ the numbers of perivascular MSPCs, pericytes, and SCF/KITL, all of which are crucial to HSC maintenance. Importantly, contraction of the endosteal and Type-H vessels also promotes myeloid skewing.^91^ Reductions in the number of arteriolar vessels are also found to cause depletion of *osteolectin*^*+*^, periarteriolar osteoprogenitors,^60^ adrenergic nerve degeneration, and compromised lymphopoiesis.^18^ Although the sinusoidal vessels have been shown to be altered to a less degree than arteriolar and endosteal vessels,^150^ aged bone marrow presents higher vascular permeability,^149^ which can be aggravated by increased RANKL/RANK level when bone remodeling is biased toward resorption (Fig. 1.4).

Taken together, studies have shown that skeletal aging alters the bone marrow microenvironment, although more evidence is needed to demonstrate direct correlations among aged bone phenotype, niche alterations, and hematopoietic functions. Of note, clonal hematopoiesis (CH) occurs with aging and is characterized by clonal growth advantages of HSCs carrying unique mutations.^151^ It is, however, unclear why mutations in certain epigenetic modifiers gain self-renewal advantages and why CH and progression to myeloid malignancies develop at different rates in patients. This likely pertains to differential regulation of the bone marrow microenvironment and, potentially, to the aged bone phenotype. Although few studies have reported the causality between skeletal aberration and clonal hematopoiesis, Kim et al. recently suggested a direct association between CH and osteoporosis.^152^ Given the strong association between the skeletal aging–inflammation and inflammation–CH axes,^153,154^ one can postulate that the bone resorption bias aggravates CH, which is the case in myelodysplastic syndromes (MDS), leukemia, and multiple myeloma, as reviewed in the following sections.

### The role of bone remodeling in regulating MDS and myeloid leukemia

MDS and myeloid leukemia (acute (AML) or chronic (CML)) are blood malignancies initiated by hematopoietic stem and progenitor cells (HSPCs), mostly due to known mutations. These malignancies are characterized by ineffective hematopoiesis due to the accumulation of immature blasts and are commonly diagnosed at an advanced age (> 65 years old), which prevents patients from receiving bone marrow transplantation and limits the options for second-line treatment.^149^ Recently, genomic testing of MDS and AML patients confirmed clonal evolution characterized by expansion of subclones that acquire unique mutations, which contributes to disease progression or MDS evolution into secondary leukemia.^155–158^ Although acquisition of age-associated somatic mutations represents a critical factor in pathogenesis, the bone marrow microenvironment, including the mutations in bone and osteolineage cells,^159–162^ is also known to drive or facilitate the malignant hematopoietic program, as studied and reviewed extensively in recent works.^163,164^ Since cellular/molecular factors involved in bone remodeling and skeletal aging impact the microenvironment and HSCs (Sections 1 and 2.1), whether altered bone remodeling during aging modulates myeloid malignancies and malignant stem cells is of great interest and needs to be better understood. Notably, both AML and MDS can develop at a young age due to certain mutations or as results of chemo- or radiotherapy. To date, it remains unclear whether growing bone characterized by rapid remodeling impacts disease initiation or progression. Arthritis that has been reported during the diagnosis of pediatric leukemia is likely associated with leukemia-induced bone lesions.^165^ However, correlating altered bone remodeling in pediatric patients who received chemotherapy^166^ to treatment outcomes and the rate of relapse may provide insight into the role of bone remodeling in pediatric blood cancer. In this section, we focus only on age-associated MDS and myeloid malignancies and review the coupling between bone remodeling and the tumor microenvironment.

#### Bone resorption bias in skeletal aging sustains the inflammatory milieu and releases growth factors required for disease propagation

An analysis of > 67 000 MDS patients showed that MDS is prevalent in people with osteoporosis, with osteoporotic men at higher risk than women in all age groups. Although this study was limited by the number of patients with concurrent MDS and osteoporosis (<0.6%) and lacked information on the disease stage and evolution, it suggested a link between structural deterioration and impaired hematopoiesis.^167^

Indeed, skeletal aging phenotypes (listed in Section 2.1) may contribute to adverse outcomes in myeloid malignancies. For example, it has been shown that RANK is overexpressed on AML cells and is linked to poor prognosis;^168^ however, whether RANK–RANKL interactions (increased in bone resorption) lead to functional consequences remains to be elucidated. Moreover, aging or radiation treatment significantly increased the number of senescent cells expressing p16 and p21,^169^ which aggravate the adverse impact of MSPC senescence in MDS and AML (Fig. 5.1), characterized by compromised MSPC osteogenic potential and augmented proinflammatory cytokine production.^170–172^ The accumulation of marrow fat during aging and the adipocyte differentiation propensity of MSPCs further negatively impact hematopoiesis.^127^ In settings of myeloid malignancies, AML patient-derived MSPCs were found to be biased toward adipogenesis with a reduction in the level of *Sox9*, which has been shown to promote the survival of leukemia blasts^173^ (Fig. 5.2). However, by examining AML engraftment in a human bone implant in mice, Battula et al. showed that the AML-engrafted bone marrow showed significantly increased, BMP-induced *Runx2*^*+*^ and *Osterix*^*+*^ osteoprogenitors (Fig. 5.3), in which the connective tissue growth factor (CTGF) from MSPCs enhanced leukemia engraftment.^174^ The inconsistency between these studies may be attributed to the experimental models used (different leukemia types and the use of bone implants), the stage of the disease, or potentially the spatial origin of cells. For example, Duarte et al. showed that MLL-AF9 AML degraded endosteal osteoblasts, the endothelium and stroma, while the vasculature in the central bone marrow was preserved and had expanded.^175^

Importantly, several growth factors/proteins released during bone resorption, such as IGF-1/2^176^, TGF-β,^177^ and BMPs^174^ have been associated with MDS/leukemia propagation. This suggests that bone resorption likely augments MDS-/leukemia-related pathways, and distinct stages of bone turnover across bone marrow cavities is expected to affect the spatial gradients of bone-derived factors to orchestrate disease progression and chemoresistance. For example, IGF-1 enforces SASP acquisition as high levels of IGF-binding proteins have been found in multiple types of senescent cells.^178^ Moreover, bone matrix and extracellular calcium released during bone resorption are potent activators of NOD-, LRR- and pyrin domain-containing protein 3 (NLRP3), which is overexpressed in MDS patients carrying various somatic mutations (*e.g., ASXL1*, *SF3B1, SRSF2*, and the del(5q) subtype) and is correlated with a poor prognosis^179,180^ (Fig. 5.4).

#### Potential roles of the reversal and bone formation phases in modulating chemosensitivity and self-renewal of leukemia cells

The coupling factors produced during the reversal stage of bone remodeling may contribute to the progression of myeloid malignancies. In the context of AML, S1P signaling is central to the initiation of leukemia, where overexpression of S1PR_3_ leads to leukemogenesis.^181^ The S1P-S1PR_3_ axis has also been found to initiate the inflammatory program and myeloid cell differentiation bias among leukemic stem cells (LSCs) from AML patients, which express high levels of S1PR_3_.^182^ Interestingly, despite its role in leukemogenesis, activation of S1PR_3_ pushed LSCs to differentiate, thereby increasing chemosensitivity^182^ (Fig. 5.5). In addition, OPN, a factor highly enriched in bone multicellular units (BMUs, the sites of bone remodeling), was found to be increased in the bone marrow of AML patients, and its level was correlated with a shortened survival.^183^ Moreover, Boyerinas et al. showed that OPN plays roles in minimal residual diseases of B-cell acute lymphoblastic leukemia by controlling cell quiescence, but whether it plays a similar role in age-related malignancies such as MDS or AML is unclear and needs to be elucidated^184^ (Fig. 5.6).

Overexpression of stromal and osteoblastic Wnt signaling has been shown to contribute to disease initiation.^159^ Moreover, the bone-forming zone participates in promoting leukemia cell expansion and maintenance of leukemia-initiating cells. For example, dormant AML cells engrafted in osteoblast-enriched regions were found to be resistant to cytarabine.^185^ MLL-AF9 murine leukemia-initiating cells localized to endosteal regions with higher ATP levels to promote cell self-renewal^186^ (Fig. 5.7). Notably, this process involved calcium ion influx, but how different levels of extracellular calcium at distinct stages of bone remodeling modulate leukemia cell expansion remains to be investigated. More recently, Galán-Díez et al. showed that AML cell proliferation depended on serotonin signaling in osteoblasts, where the oncometabolite kynurenine, a hallmark in MDS and AML patients, bound to the serotonin receptor 1B on osteoblasts and formed a vicious positive feedback loop through proinflammatory pathways.^187^ Taken together, the reversal and bone formation phases may contribute to chemosensitivity and self-renewal of leukemia cells.

#### Potential effects of coupled bone and vascular niches on the fate commitment of leukemia cells

Importantly, the arteriole/sinusoidal vascular landscape is tightly associated with bone remodeling and likely contributes to heterogeneous fate commitment of leukemia cells, as the dormancy and proliferation of leukemic stem cells have been shown to be supported by distinct subsets of perivascular niche cells and their expressed molecules. For instance, sinusoidal vessels express several adhesion molecules vital for cell dormancy and leukemia progression.^188^ Leukemia cells with high VLA-4 and CD98 expression facilitate cell adhesion to sinusoidal vessels, followed by subsequent activation of integrin signaling that promotes cell self-renewal and proliferation.^189^ In addition, in a BCR-ABL murine model of CML, the CD44/E-selectin axis was shown to be essential for proper cell engraftment, and positively associated with imatinib resistance. Blocking cell adherence using the E-selectin inhibitor GMI-1271 allowed CML-initiating cells to enter the cell cycle and thus achieve longer survival^188^ (Fig. 5.8). This outcome is consistent with the finding obtained with the MLL-AF9 murine AML model, where interactions with E-selectin enhanced chemoresistance mediated via AKT/NF-kB pathways.^190^ In addition to the perisinusoidal niche, Agarwal et al. showed that selective deletion of CXCL12 from periarteriolar Tie2^+^ MSPCs impaired the proliferation of leukemia stem cells, while CXCL12 expressed in *Prx1*^*+*^ MSPCs reinforce cell quiescence and chemoresistance^191^ (Fig. 5.9).

Taken together, these studies showed skeletal aging phenotypes contribute to an inflammatory milieu and a bias toward bone resorption, negatively regulating normal hematopoiesis and positively supporting tumor growth, while the coupling factors involved in the reversal stage and in the bone-forming niche may sustain leukemia-initiating cells. These dynamic and spatially heterogeneous processes further impact the vascular landscape and likely lead to the differential fate commitment of leukemia cells. Notably, as patients are usually diagnosed when symptoms of ineffective hematopoiesis start to appear (relatively high disease burden), the molecular and functional alterations of niche cells at this stage might have been remodeled by the malignant clones.^9,192–196^ Given that clinical MDS and myeloid leukemia are still largely driven by mutations in the HSC compartment, elucidating the niche-HSC interplay critical to early disease establishment is crucial to intercept disease progression. Specific questions that need to be addressed include the following: (i) Do emerging malignant subclones require specialized niches to survive? (ii) How do malignant stem cells outcompete the healthy HSCs. Specifically, what are the physiologic advantages of the malignant stem cells (proliferation, survival, migration, etc.)? (iii) What regulates the vicious cycle between niche-enforcing clonal expansion and expansion-induced niche alteration? The answers to these questions will provide critical insights into disease propagation and relapse, leading to development of novel interventions. Finally, it is important to consider that skeletal aging depends on sex steroid levels. Estrogen deficiency resulted in high bone turnover (activation of BMUs) and elevated serum osteocalcin level despite an overall negative bone balance.^197^ Thus, it will be important to determine whether the differential activation of BMUs and coupling factors between sexes impact disease progression.

### The role of bone remodeling in regulating plasma cell dyscrasias

Plasma cell dyscrasias constitute a spectrum of diseases, including monoclonal gammopathy of undetermined significance (MGUS), smoldering multiple myeloma (SMM), and multiple myeloma (MM). MM is the second most common hematologic malignancy.^198^ It is incurable and believed to be preceded by precursor plasma cell dyscrasias in the form of MGUS or SMM, as classified by the plasma cell burden and risk of progression to MM.^199–202^

Patients with MM often present with distinct skeletal features, including hypercalcemia and pathological fractures secondary to osteolytic bone destruction due to an imbalance in osteoblastic bone formation and osteoclastic bone resorption.^203^ Notably, initial alterations in bone are evident in patients with MGUS and SMM; however, how they relate to progression from MGUS and SMM to MM remains to be elucidated.^204^ Patients with MGUS and SMM present with a higher risk of skeletal fractures independent of decreased bone mineral density.^205^ A significantly higher fraction of SMM patients show bone abnormalities in the thoracic and lumbosacral spine, as determined by MRI.^206^ These findings suggest that alterations in bone precede the malignant disease phase. Although myeloma-induced bone diseases and the associated mechanisms have been extensively studied and reviewed,^207–210^ the cellular and molecular mechanisms involved in bone remodeling that may participate in the tumor microenvironment remain areas of interest. Specifically, aberrant bone remodeling may be critical given the high incidence (3%) of plasma cell dyscrasias,^211^ with the median age at diagnosis being 70 years.^212,213^ Therefore, here, we review the current understanding of the interactions between skeletal aging and the development/progression of MM.

#### Bone lineages and remodeling are critical for the initiation of plasma cell dyscrasias

Studies have revealed changes of skeletal cells and aging phenotypes in plasma cell dyscrasias. For example, myeloma bone disease results from bone resorption bias.^203^ An increase in the number of adipocytes in bone is prevalent in MM samples and is associated with progression of MM from asymptomatic precursors.^214–216^ Bone cells, along with the extracellular matrix components, are essential regulators of the age-related tumor niches that attract and maintain myeloma cells. Then, malignant cells aggravate osteolysis through the production or release of cytokines and growth factors, which ultimately leads to a “vicious cycle” and the development of a tumor-supportive niche.^207,217^ Thus, understanding the tumor-supportive role of bone cells in the tumor microenvironment remains an active research goal.

MSPCs have been shown to exert both tumor-supportive and tumor-inhibitory effects, depending on the cancer type or the protocols of cell isolation, culture, and characterization.^218^ In MM contexts, MSPCs have been reported to promote disease progression.^219–223^ Specifically, myeloma cells interact with MSPCs through VLA-4 and RGD peptide mechanisms or secrete fibroblast growth factor (FGF) to stimulate IL-6 expression on MSPCs, the major growth factor feeding myeloma cells.^219,220^ Wang et al. also reported that MSPCs produce survivin to protect myeloma cells from apoptosis.^221^ Additionally, MSPCs promote myeloma cell retention in the bone marrow through adhesion molecules such as CD44, VLA-4, VLA-5, leukocyte function-associated antigen 1, neuronal adhesion molecule, intercellular adhesion molecule 1, syndecan 1 and MCP-1.^224^ Moreover, MSPCs affect myeloma cells indirectly through osteoclastogennesis and angiogenesis. For instance, interactions between myeloma cells and MSPCs dramatically increase expressions of VEGF and FGF, two important angiogenic cytokines in MM.^222,223^ (Fig. 6.1) and bone marrow angiogenesis is a predictor of poor survival in patients with newly diagnosed myeloma.^225^

Adipocyte predisposition is a skeletal aging phenotype. In the context of MM, adipocytes have been shown to regulate myeloma cell migration via the actions of chemoattractive MCP-1 and CXCL12.^226^ Moreover, cell-cell contact with adipocytes is critical for supporting myeloma cells through numerous adipokines.^227^ (Fig. 6.2). For example, Leptin induces autophagy through the *Jak/Stat3* signaling pathway to promote the proliferation and prevent apoptosis of myeloma cells.^228^ Visfatin, also known as the rate-limiting enzyme in NAD+ biosynthesis from nicotinamide, is another tumor-supportive adipokine. Myeloma cells treated with its inhibitor, APO866, undergo apoptosis.^229^ In contrast, downregulation of adiponectin is detrimental in SMM and MM,^230^ as adiponectin prevents disease progression by activating p21 and p53 or activating AMPK and MAPK signaling to induce cell cycle arrest and apoptosis in myeloma cells.^231^ As bone resorption and formation may yield differential gradients of leptin and adiponectin levels (Fig. 4.3), the distinct stage of bone remodeling likely impacts MM cells through adipokine-mediated pathways.

The role played by osteoblasts in modulating MM has been controversial. The direct effect of osteoblasts was reported to induce apoptosis and cause cell cycle arrest of myeloma cells, when cultured with osteoblasts induced from the MC3T3-E1 cell line or with primary bone marrow stromal cells.^232^ In particular, Decorin has been identified as a key factor produced by osteoblasts that inhibits the proliferation and survival of myeloma cells through activation of p21^233^ (Fig. 6.3). However, osteoblasts have also been reported to produce factors, such as OPN, that sustain myeloma cells.^234^ The controversy regarding osteoblast actions may be largely attributed to their coupling with osteoclasts during the bone remodeling process. Specifically, activation of osteoblasts results in increased osteoclast differentiation. Then, tumorigenic factors are released through bone resorption; these factors include TGF-β,^235^ IGF-1^236^ and FGF, PDGFs,^237^ and BMPs.^238^ An increase in the extracellular calcium level during bone resorption also activates calcium pumps and calcium-sensing receptors, promoting myeloma cell proliferation^207,239^ (Fig. 6.4). Additionally, osteoclasts directly promote the survival and proliferation of myeloma cells by producing OPN,^234^ MIP-1a/CCL3, IL-6, Annexin II,^198^ BAFF^240^ and APRIL^241^ (Fig. 6.5). The angiogenic factors produced by osteoclasts (namely, OPN and PDGFs) or released from bone matrix via the action of osteoclast produced MMP-9 also contribute to angiogenesis and MM progression.^234,242,243^ Taken together, these results suggest that osteoblasts enriched at the modeling or remodeling sites may play differential functions, and the heterogeneity within osteoblastic lineage cells remains an important area to be addressed.

#### Senescent osteolineage cells represent a new direction for studies into the progression of MM

**Fig. 7:**
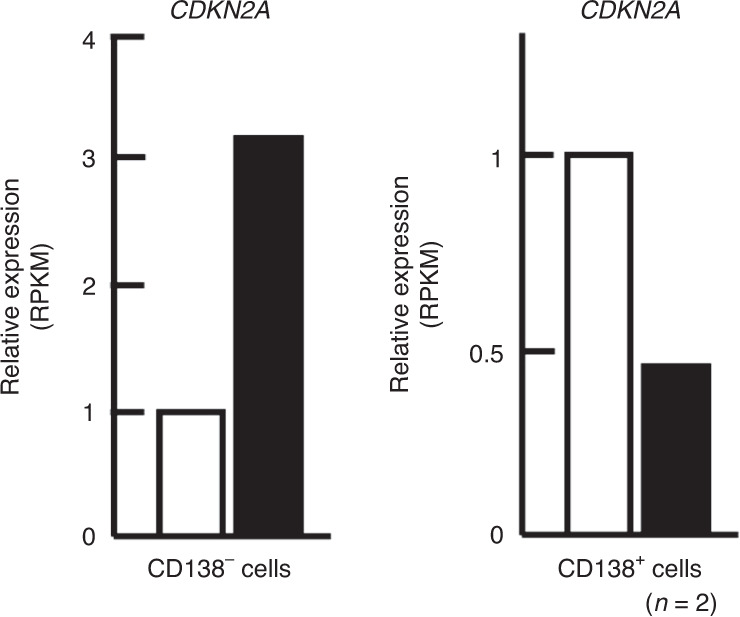
Cell senescence in SMM. The expression of the cell senescence marker gene CDKN2A in CD138^+^ myeloma cells and CD138-expressing nonmyeloma cells was measured by RNA sequencing. Black bars represent clinically diagnosed SMM patients, and white bars represent age-paired healthy donors

As shown in Fig. 6.6, cellular senescence is a hallmark of aging characterized by permanent proliferative arrest and altered gene expression patterns leading to SASP acquisition.^244^ Which cell compartment contributes to cellular senescence in the tumor microenvironment remains to be defined. Increased cellular senescence has been reported mainly in mesenchymal stromal cells,^245,246^ adipocytes^247,248^ and T cells^249,250^ in MM. In contrast to the senescence of microenvironment cells, the senescence of myeloma cells is considered to be a protective mechanism against tumorigenesis.^251^ Indeed, our preliminary data obtained from patients with SMM showed that the level of the senescence marker gene CDKN2A was decreased in myeloma cells from SMM patients but increased in nonmyeloma cells (Fig. 7), consistent with previous literature reports.^245–250^ Interestingly, we and others^135,252^ have reported that the majority of senescent cells in long bones and fracture calluses in aged mice are of the osteolineage. The senescent population negatively regulates osteoblast differentiation while inducing osteoclastogenesis,^135,252^ and therefore, likely plays supportive roles in MM progression. Several challenges and questions remain to be addressed. Further analyses are needed to elucidate the origin of senescent cells in the tumor microenvironment and to determine their direct impact on myeloma cells, but the diminished number of stromal cells in the bone marrow of MM patients makes it difficult to study cellular senescence in mature MM. In addition, an association between senescent cells and MGUS/SMM has not yet been reported. Our findings showing an increased number of senescent nonmyeloma cells in patients (Fig. 7) make it possible to isolate and examine the cellular identity, functional heterogeneity,^252,253^ and corresponding SASP factors that participate in disease progression.

## Therapeutics that target bone remodeling

Age-related myeloid malignancies and MM manifest with aberrant bone lineage cell and osteoclast functions, as reviewed in the previous sections. In particular, bone turnover plays a pivotal role in disease progression. Therefore, therapeutics allowing simultaneous interruption of malignant cell proliferation and bone destruction while enhancing bone formation are highly desired. In this section, we provide a synopsis of therapeutics that target bone remodeling and describe their effects on hematopoiesis and age-related hematological cancers.

Drugs that modulate bone homeostasis have been mainly used in managing MM. For example, proteasome inhibitors are first-line drugs for treating MM. They not only induces myeloma cell apoptosis but also inhibit osteoclastic bone resorption, increase osteoblastic bone formation, and prevent osteocyte apoptosis.^254,255^ Three proteasome inhibitors have been approved for clinical use, namely, bortezomib (approved in 2003), carfilzomib (approved in 2012) and ixazomib (approved in 2015)^256^; however, the contribution of bone homeostasis after treatment has not been extensively evaluated. DKK1 is a *Wnt* antagonist that plays a role in myeloma bone diseases mediated through RANKL/OPG pathways^257^ and contributes to the progression and relapse of MM. Therapies targeting DKK1 (BHQ880) are in development for treating MM and SMM.^258,259^ In this regard, the RANKL inhibitor denosumab has been shown to reduce DKK1 levels.^260^ Notch signaling has also been implicated in the progression of MM from precursor states through the upregulation of RANKL and alterations in the bone marrow stromal environment.^261^ To date, bisphosphonate remains one of the standard therapies to reduce the number of osteolytic lesions, ameliorate bone pain, and attenuate pathological fractures and hypercalcemia in MM. Clinically used nitrogen-containing bisphosphonates (N-BPs), such as alendronate, bind to hydroxyapatite in bone and cause osteoclast apoptosis by specifically suppressing mevalonate pathways (namely, hydroxymethylglutaryl-coenzyme A reductase) in osteoclasts.^262^ A clinical study revealed that the action of bisphosphonates translated to increased bone mineral density in patients with MGUS.^263^ However, randomized studies have evaluated the impact of bisphosphonates on SMM and reported that although bisphosphonates decrease the risk of deleterious skeletal events, progression-free survival was not prolonged.^264,265^ Overall, the connection between bone remodeling after these treatment and disease progression remain to be characterized.

Although the aforementioned drugs have not been used in clinical practice for age-related myeloid malignancies, increasing evidence has suggested their beneficial effects in controlling tumor burden by reversing bone-destructive phenotypes and potentially auxiliary mechanisms. Here, we review the impact of PTH fragments or analogs and bisphosphonates on hematopoiesis and age-related myeloid malignancies in preclinical settings. The mechanisms of action of PTH on bone remodeling were reviewed in detail by Wein and Kronenberg.^266^ Notably, PTH also profoundly impacts hematopoiesis. Intermittent treatment with PTH for 4 weeks expanded the HSC (LSK) population. It also conferred a self-renewal advantage to HSCs and increased the reconstitution capacity after myeloablative conditioning.^41^ In addition to regulating normal HSCs, PTH has been shown to lead to different outcomes in murine models of CML (BCR-ABL1) and AML (MLL-AF9). Specifically, intermittent infusion of PTH prolonged animal survival, as increased bone turnover induced by PTH caused TGF-β1 release from the bone and suppressed the BCR-ABL1 leukemic stem cells. In contrast, PTH has been found to accelerate the expansion of MLL-AF9 cells. Notably, MLL-AF9 leukemia cells lack the TGF-β1 receptor, suggesting that additional mechanisms are involved in bone remodeling that govern cell expansion.^8^ More recently, based on the findings indicating disruptive osteogenesis and increased angiogenesis in MDS,^9^ abaloparatide, a PTH/PTHrP analog, is being tested in combination with bevacizumab (an anti-VEGFA antibody) in a Phase I clinical trial as a treatment for MDS patients (NCT03746041). In addition to that of PTH, the role of bisphosphonates in leukemia remains unclear. Recent work from Chiarella et al. showed that bisphosphonate (zoledronic acid, ZOL) inhibited MLL-AF9 leukemia cell proliferation in vitro.^267^ ZOL may mediate hematopoiesis and leukemia cells indirectly by modifying the bone marrow microenvironment, including increasing the endosteal BMP2 and BMP6 signaling,^268^ decreasing the level of circulating angiogenic factors,^269^ inhibiting angiogenesis^,^^270^ and increasing antitumor immunity.^271^ Interestingly, ZOL has been reported to cause the expansion of HSPCs^272^ and increase the expression of self-renewal genes (*Bmi1*) in *Lin*^*-*^ cells^268^; however, its effect on the malignant stem cells has not been evaluated. Therefore, identifying the optimal dose regimen for realizing its antitumor effects without increasing the risk of expanding leukemia-initiating cells will be a critical step toward treatment and worthy of further investigation.

## Conclusions and future perspectives

To date, research on stem cell niches has been largely focused on endosteal and perivascular niches. However, bone marrow cavities, which are characterized by distinct stages of bone remodeling, shape heterogeneous clonal responses of HSCs and acute myeloid leukemia cells. Through research, information on how the cellular constituents and molecular pathways at each stage of bone turnover may communicate with the hematopoietic compartment continues to emerge. In the steady state, the resorption stage shapes a microenvironment that may enhance myeloid bias and cell motility. In contrast, the bone formation stage may be more supportive of lymphopoiesis and stem cell maintenance. The reversal stage may involve mechanisms that reinforce immune privilege, self-renewal-based cell expansion and motility. Importantly, bone remodeling is substantially altered in conjunction with skeletal aging. The subsequent formation of a proinflammatory and senescent milieu and bone resorption bias constitute tumor-supportive programs. In addition, factors involved in resorption (e.g., RANKL and growth factors), reversal (e.g., S1P and OPN), and bone formation stages (e.g., Wnt and ATP) also act differentially on malignant cells. As increasing evidence suggests the interdependency of bone and bone marrow in regulating hematopoiesis, substantial effort is needed to better understand the impact of bone remodeling on hematopoietic functions and age-related hematologic disorders.

There are several key questions to be answered. For example, in what scenarios does bone remodeling act as a master (upstream) regulator, and when is it a responder? As bone homeostasis is regulated by systemic factors (e.g., hormones and daily weight-bearing activities) that are not necessarily dependent on hematopoiesis, bone remodeling may serve as an upstream regulator that likely superimposes an additional dimension of heterogeneity within local hematopoietic microenvironments. As illustrated in Fig. 1–4, how the stem cell niche and HSCs respond to bone remodeling remains to be explicitly demonstrated; specifically, the functional impacts of spatiotemporal alterations in endothelial cell subsets, perivascular stromal cell subsets, the MK-osteoblast-osteomac units, the SNS, immune subsets, and chemical constituents (e.g., OPN, coupling factors, MMPs, calcium, and OSM gradients) need to be determined. Importantly, skeletal aging and disease-induced changes in bone remodeling largely involve the inflammatory and senescent microenvironment and likely lead to disease aggravation. The major players that contribute to inflammation and cellular senescence and their interplay with bone quality remain an appealing line of inquiry in many age-related hematologic malignancies. These ambiguities may be addressed via advances in high-resolution live-animal imaging and single cell next-generation sequencing.^273^ Finally, clinically approved osteoporotic treatments, such as PTH analogs and bisphosphonates, may exert effects in addition to those exerted on the bone cells due to coupled bone resorption and formation processes, as illustrated in Fig. 1–3. In a scenario where bone turnover is frequent (an increased number of regions show a mixture of bone formation and resorption processes) either due to osteoporotic treatment or gender-specific skeletal aging, it may involve stem cell self-renewal in normal and malignant hematopoiesis. Understanding the role played by bone remodeling in modulating the bone marrow microenvironment and hematopoietic cells will provide insights useful for the clinical intervention of age-related hematological malignancies using bone-targeting drugs.
